# Benign Cystic Peritoneal Mesothelioma Revealed by Small Bowel Obstruction

**DOI:** 10.1155/2016/6728160

**Published:** 2016-03-15

**Authors:** Kaimba Bray Madoué, Moifo Boniface, Edzimbi Annick Laure, Herve Pierre

**Affiliations:** ^1^Surgical Service, Renaissance Hospital of N'Djamena, N'Djamena, Chad; ^2^Faculty of Medicine and Biomedical Sciences (FMBS), The University of Yaounde I, Yaounde, Cameroon; ^3^Radiology Service, Renaissance Hospital of N'Djamena, N'Djamena, Chad

## Abstract

Benign cystic peritoneal mesothelioma is a rare tumor which frequently occurs in women of reproductive age. Abdominal pain associated with pelvic or abdominal mass is the common clinical presentation. We report the case of a 22-year-old woman with a pathological proved benign cystic mesothelioma of the peritoneum revealed by a small bowel obstruction and a painful left-sided pelvic mass with signs of psoitis. Contrast enhanced abdominal CT-scan demonstrated a large pelvic cystic mass with mass effect on rectosigmoid and pelvic organs. The patient underwent surgical removal of the tumor. Pathological examination revealed the diagnosis of benign cystic mesothelioma of the peritoneum. The outcome was excellent with a 12-month recoil.

## 1. Introduction

Benign cystic mesothelioma (BCM) of the peritoneum is a rare intra-abdominal tumor with a strong predilection for the peritoneum of pelvic organs [1]. This lesion occurs most frequently in women during their reproductive years and is associated with a history of previous abdominal surgery, endometriosis, or pelvic inflammatory disease [2–4]. We report the case of a 22-year-old woman with a pathological proved benign cystic mesothelioma of the peritoneum revealed by an intestinal obstruction.

## 2. Case Report

Ms. KA, 20 years old, was referred to surgical consultation of N'djamena Renaissance Hospital (NRH) for diffuse abdominal pain, nausea, and vomiting evolving for four days.

She had a medical history of premature delivery in 2014, followed by a puerperal infection. Since then the patient complained of constipation, gurgling, and abdominal bloating.

On clinical examination, her temperature was 36.6°C, her blood pressure was 99/55 mmHg, and her general condition was deteriorated with weight loss (36 kg) and pale mucous membranes. Physical examination demonstrated a painful left-sided abdominal mass with signs of psoitis; the rest of abdomen was distended and tympanic on percussion. The pelvic examination was unremarkable. We concluded on an intestinal obstruction probably due to left-sided abdominal mass of unknown origin.

**Figure 1:**
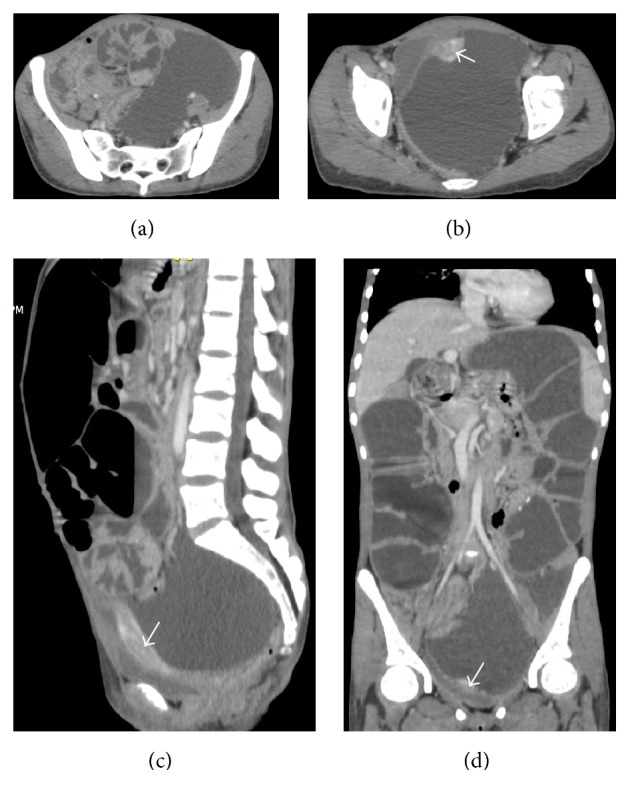
Contrast enhanced abdominopelvic CT-scan (portal phase). Axial (a, b), sagittal (c), and frontal (d) images demonstrated a large cystic pelvic mass extended to the left iliac fossa, with enhanced regular thin wall. There is mass effect on the uterus (arrow) and distended small bowel loops.

**Figure 2:**
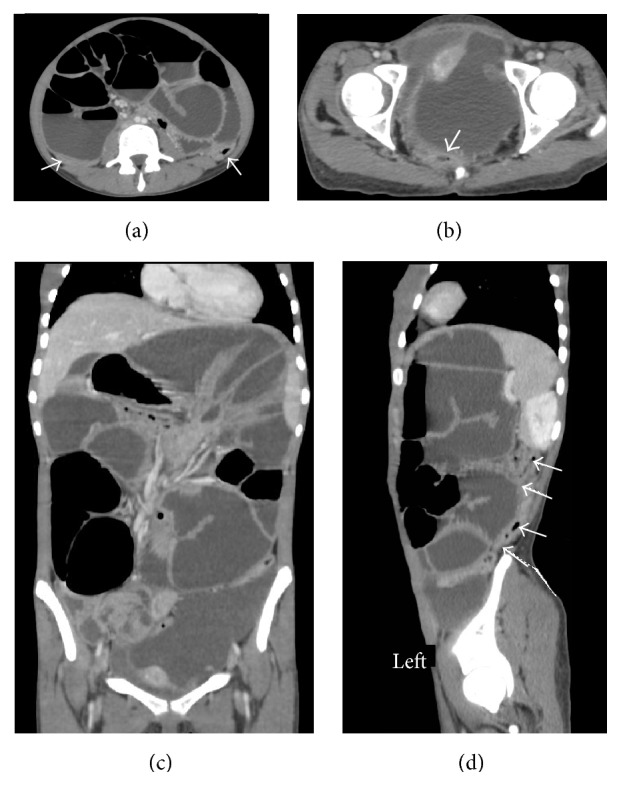
Contrast enhanced abdominopelvic CT-scan (portal phase). Axial (a, b), frontal (c), and left parasagittal (d) images demonstrated distended small bowel loops with air fluid level and mass effect on the large intestine (arrow) which is completely flat.

*Laboratory Tests Were Unremarkable*. A contrast enhanced abdominopelvic CT-scan demonstrated a large left-sided abdominopelvic thin-walled cystic mass of 103 mm × 122 mm, with mass effect on the large intestine and pelvic organs (Figure 1). Moreover, CT-scan confirmed small bowel loops distension with air fluid levels; the intestinal wall was normal; there was no transitional zone as the obstruction was the result of large intestinal compression by that abdominopelvic mass (Figure 2).

**Figure 3:**
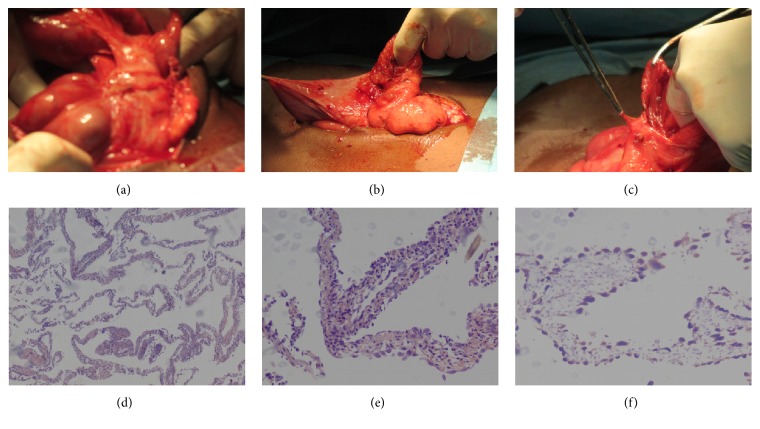
Surgical view (a, b, c) showing adhesions of the peritoneal cyst on intestine and mesentery. Pathological examination images. Hemalun-erythrosin-safran coloration (d, e) and immunohistochemistry with anticalretinin (f), a tangled maze of thin membranes, bordered on each side by a coating or slightly hyperplastic mesothelial (CK7 +, calretinin +).

The diagnosis of small bowel obstruction due to mass effect by a large cystic benign-like abdominopelvic mass was retained. The patient underwent an emergency surgery during which a cystic mass of the Douglas pouch was revealed. The tumor was adherent to small and large bowel and makes dissection and resection very difficult (Figure 3). Complete resection of the lesion was performed. The rest of the digestive tract was enveloped in a membrane.

Pathological examination (Figure 3) of the cyst wall showed a tangled maze of thin membranes, bordered on each side by a coating or slightly hyperplastic mesothelial (CK7 +, calretinin +). The mesothelial tissue underneath is fibroedematous and contains a discreet inflammatory infiltrate polymorph.

## 3. Discussion

Benign cystic mesothelioma (BCM) of the peritoneum was primarily described by Mennemeyer and Smith [1]. It is a proliferation of mesothelium cells of the peritoneum, with a predilection for the pelvic viscera [2]. About 150 cases have been reported in the literature [3].

The pathogenesis is controversial. The close relationship with inflammation, a history of prior surgery, endometriosis, or uterine leiomyoma suggests that it is probably a peculiar peritoneal reaction to chronic assault, with mesothelial cell entrapment, reactive proliferation, and cystic formation [4].

It frequently occurs in women of reproductive age, with a history of surgery, pelvic inflammatory disease, or endometriosis [5]. Abdominal pain, tenderness, and distension, usually in association with a pelvic or abdominal mass, are the common presenting features [6]. Our case was attended to in our hospital with signs of intestinal obstruction and pelvic mass. This makes our case unique in terms of presentation.

The correct diagnosis can be made by histopathological examination in conjunction with immunohistochemical and ultrastructural evaluations [7].

Electron microscopy shows the characteristics of mesothelial cells-slender microvilli on the luminal surface of the cells, desmosomes, intracytoplasmic intermediate filaments, endoplasmic reticulum, and dilated mitochondria. Immunohistochemistry shows strong staining for cytokeratin in the cyst linings and for vimentin in the subepithelial cells [6].

Differential diagnoses are numerous, including cystic lymphangioma, mucinous cystadenoma, cystic teratoma, cystic mesothelioma, mullerian cyst, epidermoid cyst, tailgut cyst, bronchogenic cyst, cystic changes in a solid neoplasm, pseudomyxoma retroperitoneal, perianal mucinous carcinoma, pancreatic pseudocyst, lymphocele, urinoma, hematoma, cystadenoma of mesonephric origin, and cavernous hemangioma Dinesh [8].

It is agreed that surgery is the only effective treatment, with complete removal of the cystic lesions as the mainstay of treatment and the only chance for avoiding local recurrence [9].

The tumor has been reported to include various parts of the serosa of the bowel and sometimes spreads to liver, spleen, and pancreas. Rarely the tumor may present as free floating pelvic cysts [6].

The prognosis is usually good. The 5-year survival is 100%. So far, only one malignant transformation has been reported [10]. The main complication thus resides in the high potential for recurrence causing significant morbidity [5]. Hyperthermic intraperitoneal chemotherapy (HIPEC) may be an alternative to reduce or avoid this recurrence.

## 4. Conclusion

Benign cystic peritoneal mesothelioma is a rare cystic mesothelial lesion that occurs predominantly in reproductive aged women. Preoperative diagnosis is very difficult, and the final diagnosis always requires pathological analysis. Surgery is the only effective treatment. The prognosis is usually good.
